# Inunction of Creasote in Malarial Fevers

**Published:** 1899-07-22

**Authors:** 


### INUNCTION OF CREASOTE IN MALARIAL FEVERS.

Major A. O. Fitzgerald,[1] R.A.M.C., records a series of cases in which he used inunction of creasote in the treatment of malarial fevers. Pure beechwood creasote, in doses of 15 to 20 minims for a child of one year, or 30 to 60 minims for an adult, was mixed with an equal quantity or more of olive oil, and rubbed for from five to ten minutes over the abdomen, axilla, and sides. The action in relieving the fever, delirium, and other symptoms seems in some cases to have been remarkably rapid, and, as Dr. Fitzgerald calls it, "specific." It is odd, but very interesting.

[1] Brit. Med. Jour., July 15.

